# Influence of Mind Wandering and Increased Attentional Demands on Multitasking and Implicit Learning

**DOI:** 10.5964/ejop.14605

**Published:** 2025-02-28

**Authors:** Cameron G. Wittschen, Christopher A. Was

**Affiliations:** 1 Department of Psychology, Kent State University, Kent, OH, USA; Victoria University of Wellington, Wellington, New Zealand

**Keywords:** implicit learning, multitasking, mind wandering

## Abstract

The goal of the current study was to replicate resent findings that suggest mind wandering is associated with impaired explicit learning but not implicit learning, and to extend those finding by investigating whether explicit learning is impaired under attentional load, but implicit learning is not. We used a sequential learning task, specifically a serial reaction task (SRT), to determine if mind wandering would interfere with learning a task that does not require attentional resources (implicit learning). Participants completed the serial reaction time task while watching a 13-minute video lecture. At the end of the video participants answered 10 multiple-choice questions regarding the content presented in the video. At specific intervals during the task, participants responded to mind wandering probes. The probes required participants to report where their attention was in the moments before the probe appeared. Implicit learning was measured by decreased reaction time over the course of several blocks of trials of the SRT. In two experiments, it was observed that participants implicitly learned a sequence of 12 items, regardless of their performance on the multiple-choice item regarding the concurrent video content. Even those who appeared to actively engage with the video and performed well on the multiple-choice questions showed improved performance on the implicit learning task (SRT). These results suggest implicit learning can occur when one is engaged in a concurrent explicit learning task.

Picture yourself in a packed lecture hall where the professor is illustrating how to analyze an extensive set of data from a longitudinal study. The analysis involves multiple complex components, and you diligently follow along, taking notes on your computer for later review. The room hums with the dull clatter of keyboards as students diligently take notes, their screens going back and forth between slides containing equations and graphs previously projected onto the board. As you’re jotting down a specific equation and attempting to comprehend your professor’s explanation of its significance to the analysis, you notice the person to your right reading a text message that just appeared on their screen. Believing in their multitasking abilities, they continue copying notes, listening to the lecture, and occasionally responding to the text conversation when a notification appears on their phone. To your left, you notice someone who looks inattentive and is doodling relentlessly in their notebook. Clearly, the person to your left is not paying attention to the lecture and seems to be adrift in their own thoughts. Considering the additional demands of responding to the texts while trying to encode the presented information, one would expect the student attempting to multitask will not perform well on an upcoming examination of the covered material. Similarly, your classmate that is mind wandering, will also likely not perform as well as they could if their attention was focused on the lecture.

Substantial evidence exists that multitasking impairs task performance, especially when one of the tasks is difficult (e.g., Adler & Benbunan-Fich, 2012; 2015), There is also a plethora of evidence that mind wandering (e.g., Randall et al., 2014; Thomson et al., 2014) impairs performance, yet recent evidence suggests that some learning may occur even when one is mind wandering (Brosowsky et al., 2021). Brosowsky et al. (2021) used a serial reaction time task (SRT) with implicit- and explicit-learning conditions to evaluate whether implicit learning occurs even when participants report mind wandering. Their results suggest that the degree of mind wandering was negatively associated with learning in the explicit condition (i.e., greater mind wandering led to poorer performance) but not in the implicit condition.

They interpreted their results as suggesting that mind wandering is associated with impaired explicit learning, but not impaired implicit learning and that that explicit learning is impaired under attentional load, but implicit learning is not. This final claim—implicit learning is not impaired under attentional load—was not directly tested. Brosowsky et al. (2021) did not have participants attempt to complete the SRT while concurrently completing an explicit learning task, but rather participants in the explicit condition were told about the repeating pattern in the SRT and to try to learn the pattern. The aim of the current study was to replicate the finding that implicit learning occurs during mind wandering, and to extend Brosowskyet al.’s findings by evaluating whether implicit learning can occur while a concurrent attention demanding (explicit learning) task is also being performed.

Brosowsky et al. (2021) used the SRT, an implicit task, to assess whether participants exhibited implicit learning when reporting instances of mind wandering. Before delving into the specifics of this implicit learning task, it is crucial to fully understand the distinction between the underpinnings of implicit learning and its counterpart, explicit learning. Implicit learning refers to a type of learning that occurs under the absolute threshold when information is obtained by the individual unconsciously and not intentionally (Kaufman et al., 2010). Several investigations of implicit learning suggest that implicit learning functions automatically when attention is directed towards a stimulus at a relatively low-level perceptually, without the dedication of executive attention resources (Cowan, 1995; Frensch & Miner, 1994). Explicit Learning, on the other hand, can be defined as a type of learning in which the individual deliberately and intentionally obtains information (Stadler, 1997). When explicit learning occurs, information is actively being sought out. Graf and Schacter (1985) state that “explicit memory is revealed by intentional recollection from a specific previous episode, whereas implicit memory is revealed when performance on a task is facilitated without deliberate recollection from a specific learning episode” (pp. 501). Put differently, explicit learning is purposeful acquisition of knowledge, while implicit learning is unintentional or incidental acquisition of information. Regarding the difference between explicit and implicit learning tasks, explicit learning tasks are organized to offer precise instructions and an emphasis on conscious comprehension whereas implicit learning tasks are defined by the indirect and experiential nature, which features minimal explicit guidance and feedback (Heuer et al., 2001).

In the experiment conducted by Brosowsky et al. (2021), participants completed a SRT task during which they were presented with a sequence of visual stimuli in a predetermined sequence on a computer screen and were instructed to respond to the stimuli by pressing corresponding buttons on a response box. Participants were instructed to respond quickly without sacrificing accuracy. Participants were assigned to one of two groups. The “explicit” group were informed that the stimuli were to be presented in a predetermined sequence and instructed to attempt to memorize the sequence. Those in the “implicit” group did not receive any information about the sequence and were simply instructed to respond to the stimuli by pressing the corresponding keys. While completing the SRT, participants were also required to respond to several mind wandering probes during which they indicated the degree to which they were mind wandering during the task. Following the presentation of the sequence in several blocks of trials participants completed two tasks to determine if they had explicitly learned the sequence: one required to participants to attempt to recreate the stimuli sequence and the second required participants to recreate it backward.

Based on the results of the experiment, Brosowsky et al. (2021) reported that implicit learning does in fact occur while mind wandering is occurring. Put differently, in the implicit condition, participants’ responses in the during the SRT became faster over the course of the experiment no matter the degree to which they reported mind wandering whereas performance of those in the explicit condition suffered as the degree of mind wandering increased.

## Mind Wandering

While the relationship between mind wandering and implicit learning may be complex, there are theoretical explanations of the Brosowsky et al. (2021) results. Mind wandering is defined as a shift from an ongoing activity to task-unrelated thoughts (TUTs). These TUTs are extremely common. Extant research suggests that we mind wander 30%–50% of the time (Levinson et al., 2012; McVay & Kane, 2012). It is also clear from the extant literature that TUTs typically lead to decrements in performance in the ongoing activity (McVay & Kane, 2012; Unsworth & McMillan, 2013). Smallwood and Schooler (2006) stated that mind wandering demands attentional (executive control) resources. This demand on executive control draws resources away from the task at hand without proper metacognitive monitoring. However, the extant literature has focused on how TUTs impair performance on explicit learning and other tasks in which attentional resources are required. Little to no research has focused on the impact of mind wandering on performance on implicit learning tasks prior to Brosowsky et al. (2021). Extant literature suggests that implicit learning, specifically implicit sequence learning, is not working memory or attention resource demanding (Janacsek & Nemeth, 2013) and thus allows for mind wandering to occur during the implicit task. Put differently, there are more working memory resources allowed for mind wandering because implicit learning is not resource demanding.

In summary, participants can implicitly encode information when their cognitive resources aren’t exclusively focused on memorizing the pattern. Based on Brosowsky et al. (2021)’s findings, it can be inferred that there are available cognitive resources during instances of implicit learning. This surplus capacity may allow for mind wandering during implicit learning, thus the potential for success in implicit sequence learning tasks even if mind wandering is occurring, as evidenced by the Brosowsky et al.’s findings in the data. The question that remains unanswered is whether the cognitive resources that are not called upon for the successful completion of an implicit sequence learning task can be recruited for the successful completion of a concurrent explicit learning task. Put differently, can an individual successfully multitask if one task is an implicit learning task and the other an explicit learning task?

## Multitasking

Multitasking is defined as a shift in attention to perform several independent but concurrent tasks (Adler & Benbunan-Fich, 2012). Multitasking is considered to have two key components: task independence and performance concurrency (Benbunan-Fich et al., 2011). Independence requires that ongoing tasks are self-contained, and the notion of concurrency requires that multiple tasks are carried out with some temporal overlap. At the personal level, multitasking involves one dividing their mental resources among various tasks. The effectiveness of managing multiple tasks is influenced by the nature of the tasks and the available mental resources (Spink et al., 2008; Waller, 1997). To understand how multitasking affects an individual, it is also important to consider an individual’s environment when they are multitasking. Multitasking is a phenomenon that occurs in various settings: at the workplace, in classrooms, while performing household chores at home, and even during focused study sessions. In an investigation of multitasking in college students, Calderwood et al. (2014) measured how often and for how long college students engage in media multitasking while doing schoolwork outside of class. Calderwood established that on average, students faced 35 distractions in a 3-hour independent study session, spending roughly 26 minutes (around 14% of the study time) on these distractions. The results of Calderwood et al. highlight the significance of multitasking in students’ lives and underscores the expectation for students to engage in multitasking for academic success. Based on the findings from Calderwood et al.’s research, when students multitask at elevated levels, it is foreseeable that errors may occur while attempting to complete various tasks simultaneously. Regarding the connection between accuracy and multitasking, Junco (2012) investigated the impact of non-academic social media use in a classroom setting on students’ overall semester GPA. Junco found that “indeed, frequency of multitasking with certain information and communication technologies (Facebook and text messaging) were negatively predictive of overall semester GPA” (Junco, 2012). Junco’s results suggest that students who multitask during classroom study are less likely to achieve academic success. This is likely attributed to the challenge students face in dividing their attention among various stimuli, often giving excessive focus to unrelated and unimportant activities like texting and Facebook messaging. The results also suggest that students are better served by concentrating on a single attentional demand, as multitasking tends to result in poorer performance.

If we consider multitasking from a cognitive resource perspective (e.g., attention, working memory), attempting to complete more than one task at the same time should require more executive control resources, if the tasks are explicit in nature. However, if Brosowsky et al.’s (2021) conclusions that implicit learning is not resource demanding are correct, then one might hypothesize that it is possible to implicit learn while one is engaged in completing a concurrent explicit task.

## Current Study

In the current investigation, we aimed to replicate the observation that implicit learning may occur during mind wandering. Furthermore, we hope to expand on Brosowsky et al.’s (2021). research by exploring whether implicit sequence learning can occur concurrently with a demanding attention task (explicit learning). In two experiments, participants were presented with a SRT task paired with an unrelated video lecture. The SRT task is a common task used amongst various fields of psychology (Robertson, 2007). A SRT is frequently used to measure implicit sequence learning (Schwarb & Schumacher, 2012) and focuses on overcoming obstacles associated with explicit learning of sequence information (Lashley, 1951). Thus, the SRT is appropriate for the implicit learning demand for this experiment. In our SRT task, there were four response options corresponding to colored keys on a keyboard. On each trial an X in one of four colors (blue, green, red, or yellow) appeared on the screen and participants had to press the corresponding key as quickly as possible. The stimuli occurred in a predetermined sequence about which the participants were not informed. In this replication and extension, we hypothesized that implicit sequence learning (in the SRT) would occur even when participants report mind wandering and a secondary task with explicit attentional demands (video lecture) is administered concurrently with the implicit task. The results of this replication and extension will provide valuable insights into human multitasking capabilities and understanding of implicit sequence learning.

## Experiment 1

### Method

#### Participants

Ninety-four undergraduate students enrolled at a large Midwestern research university participated in exchange for course credit. Upon arriving at the laboratory, participants were asked to read and sign an informed consent form. All ethical guidelines of the American Psychological Association were followed in the interactions with participants.

#### Apparatus

Participants performed all measures on PCs with SVGA monitors and standard keyboards. Programming of all tasks was completed with E-Prime 3.0 software (Schneider et al., 2002). E-Prime controlled the stimulus presentation, timing, and data collection.

#### Materials and Procedures

##### Serial Reaction Time (SRT) Task

The primary task was a serial reaction time (SRT) task with four response options, each corresponding to a colored key on a standard QWERTY keyboard. The keys used in the task were colored as follows: C, red; V, yellow; N, green; and the M, blue. The computer display presented a colored X in 64-point font in the center at the bottom quarter of the screen. The X was displayed in one of the four colors against a black background and participants were instructed to press the corresponding key as quickly and as accurately as possible. The X was presented for 500 ms with a 750 ms blank screen between each presentation.

The experiment was like a typical SRT paradigm. Participants completed 50 practice trials of the serial reaction time task (SRT) to familiarize them with the task and the location of colored keys on the keyboard. During the practice, the sequence of colored Xs was randomized. Following the practice trials, participants completed 13 blocks of a repeating 12-element sequence. The entire task consisted of 13 blocks of 48 trials (4 repetitions of the 12-element sequence per block) for a total of 624 trials. The sequence was designed to adhere to a 12-element refined conditional sequence (Pasquali et al., 2019) such that each target appeared three times per sequence and each transition occurred with the same frequency. Each colored X appeared on the screen for 500 ms with or until the participant responded. In between each sequence the space at which the X appeared was blank for 750 ms.

#### Video Lecture

**Figure 1:**
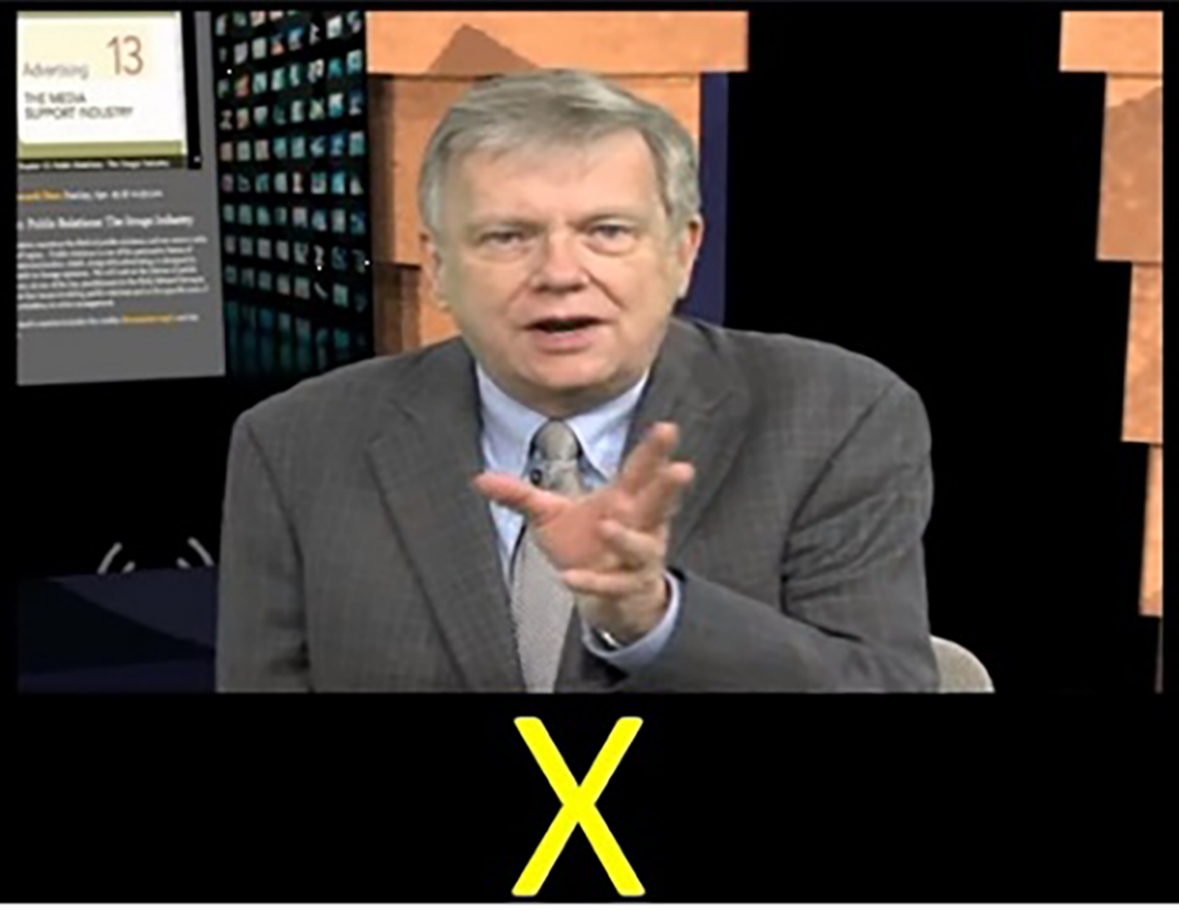
Experimental Task Display Presentation

Concurrent with the SRT participants were presented with a 13-minute video lecture. The video was presented in the center on the top half of the screen. Figure 1 presents an example of the stimuli presentation. The video used in the study was an authentic video featured in a fully online course. The content of the video was an introduction to public relations. The video was recorded in an HD studio and featured a presenter delivering a lecture directly to the camera. The timing of the SRT (stimulus duration and interstimulus interval) were set to ensure that the SRT and the video would end simultaneously.

#### Mind Wandering Probes

As the participant completed the serial reaction task and watched the video simultaneously, a series of 8 mind wandering probes appeared on the screen. When a probe arose, the video and task would pause, and the probe would ask the participant where their attention was focused in the moment just prior to the probe. The options were A – the video, B – the serial reaction time task, or C – something else. The 8 probes were administered after Blocks 2, 3, 4, 6, 8, 10, 12, and 13 of the SRT.

#### Explicit Learning Test

After the 13-minute video lecture ended and the 13 blocks of the serial reaction time task were completed, participants answered 10 multiple choice questions regarding the content presented in the video. Participants were asked to answer the 10 multiple choice questions to the best of their ability, and the scores were recorded.

#### Procedures

Upon arriving at the laboratory, participants were asked to read and sign an informed consent form. After consenting, the participants were seated at a desk with a computer, a set of headphones, and a keyboard that had colored stickers on the C, V, N and M keys. Participants were informed that they would be completing a split screen task. The instructions informed participants that at the top of the screen a video would be displayed and that they should pay special attention to the information presented in the video. Instruction also informed participants that at the bottom of the screen the letter X would appear in one of the four colors and to press the corresponding key on the keyboard as quickly and accurately as possible. They were also told that during the experiment a question regarding where their attention was focused would appear and to answer the questions honestly. Finally, they were informed about the 10-item multiple choice quiz regarding the content of the video and to answer the questions to the best of their ability.

### Results

#### Mind Wandering

Response variability to the eight mind wandering probes was extremely limited. Only eight participants responded to the probes with either *the serial reaction task* or *something else.* All other participants responded to each response probe with *the video*, therefore, mind wandering probes are not included in any analyses.

#### Multiple Choice Questions

The mean proportion correct was .43 with a standard deviation of .23. Scores ranged from .00 to 1.00. Although low, a two-tailed, single-sample *t*-test indicated that performance on the quiz was above chance performance of .25, *t*(92) = 7.73, *p* < .001, *d* = .80.

#### Serial Reaction Task (SRT)

**Figure 2:**
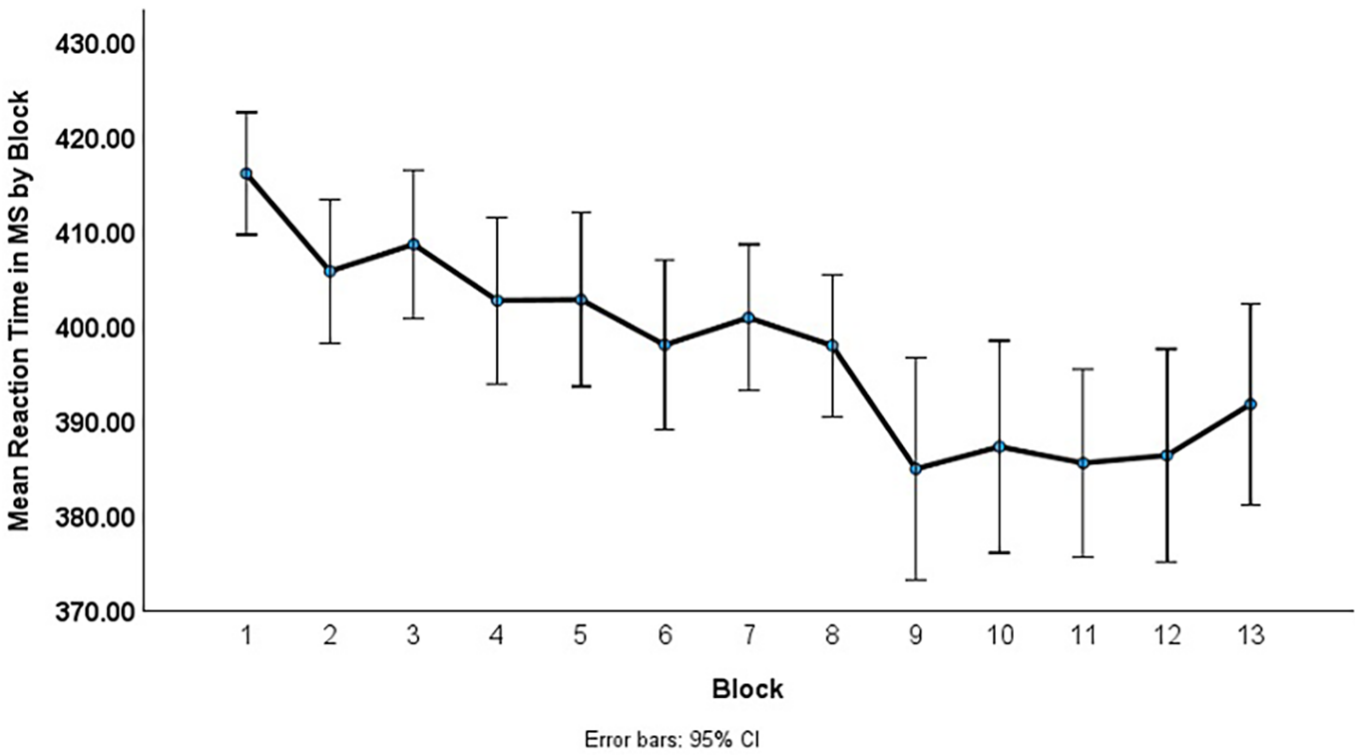
Mean Reaction Time by Block of Trials

Figure 2 displays the mean reaction time (RT) for correct responses to the 13 blocks of trials. The mean RT improvement between Blocks 1 and 2 likely reflects practice effects. To measure rate of performance improvement, mean RTs per block were calculated and then fit to three different functions (linear, exponential, and power) across Blocks 2–13. Although the exponential and power functions were both significant, the linear function provided the best fit to the data *F*(1,1074) = 28.91, *R^2^* = .03, *p* < .001. Although small, the *R^2^* value of .03 indicates that 3% of the variance in reaction time is accounted for by time (blocks) and a paired-samples *t*-test indicates a significant increase in reaction time from the first block of trails to the last block of trials, *t*(92) = 1.31, *p* < .001, *d* = .51. Although R-squared provides an estimate of the strength of the relationship between the model and response variable, it does not provide a test of the significance of the relationship, nor a formal test of the hypothesis of change over time. The F-test does provide a test of the statistical (inferential) relationship. Low R^2^ values do not mean that the predictors are not statistically significant and in the case of curve estimations of change in response times, inherent high within subject high variability in response times make it difficult to accurately predict criterion values based on the independent variable. In the current data, parameter estimates indicate an intercept of 410.77 and *b* = -2.09 (*t* = -6.98, *p* < .001) indicating that participants’ reaction time decreased significantly over the 12 blocks of trials. This decrease in reaction time suggests that participants implicitly learned the sequence of the presentation of the colored X stimuli.

To verify that performance improvement was not only due to practice effects, correlations were also calculated between RT differences between Blocks 2–3 with RT differences between Blocks 12–13. The correlation was not significant (*r* = .06, *p* = .61), suggesting that although practice effects could not be completely ruled out, the linear fit parameters are valid for capturing overall performance improvement across blocks.

Due to the range of performance on the multiple-choice items, to determine if implicit learning of the sequence learning occurred in conjunction with learning of the video content a linear function slope (*b*) was calculated for each individual. We calculated a correlation with the linear function and the proportion correct on the multiple-choice items. The correlation was not significant (*r* = .11, *p* = .29) suggesting that whether or not participants were paying attention to the video lecture they were implicitly learning the sequence presented in the SRT.

### Discussion

The results of Experiment 1 suggest that participants implicitly learned the 12-items sequence. This was the case no matter the level of learning of the video content. Put differently, even those participants who paid greater attention to the video and scored well on the multiple-choice questions demonstrated improved performance on the SRT. Although the evidence is compelling, there are two aspects of Experiment 1 that need to be addressed. First, we did not change the 12-item sequence to a random sequence in a block near the end of the experiment. Changing the sequence to a random presentation of the stimuli is common in SRT tasks. Changing a later block to a random sequence provides evidence that implicit learning of the repeated sequence has. If participants RTs slow during the random sequence, that provides further evidence for learning of the patterned sequence learning. Second, many researchers include an explicit sequence generation task at the end of the SRT. In this generation task participants are required to attempt to recreate the sequence presented. If participants are unable to recreate the sequence and they show significant decrease in reaction time during the SRT this suggests that improvement during the SRT is implicit and not due to explicitly remembering the sequence. Experiment 2 includes both blocks of random trials at the end of the SRT and a sequence generation task following the SRT.

## Experiment 2

### Method

#### Participants

One hundred and eleven undergraduate students enrolled at a large Midwestern state university participated in exchange for course credit. Upon arriving at the laboratory, participants were asked to read and sign an informed consent form. All ethical guidelines of the American Psychological Association were followed in the interactions with participants.

#### Materials and Procedures

The experimental procedure was the same as that of Experiment 1 with two exceptions. First, the serial reaction time task consisted of 15 instead of 12. Blocks 1–12 used the same repeated 12 item sequence as in Experiment 1. Block 12 was followed by a 13^th^ non-sequence random block of trials, a 14^th^ block of sequence trials and a 15^th^ block again with a completely random sequence. This design resulted in 720 trials and as the timing of trials was the same as Experiment 1, participants may have completed some of the SRT trials after the video had ended. If this was the case for any participants, Blocks 13–15 may have been completed without the explicit attention task of the video.

Second, after the 13-minute video lecture had ended and the 15 blocks of the serial reaction time task were completed, participants were asked to generate the sequence presented in Blocks 1–12 and 14 of the serial reaction time task (sequence generating task). The accuracy of the participant’s ability to generate the correct sequence was recorded. After the participants attempted to enter the 12-item sequence, they responded to the multiple-choice questions regarding the content presented in the video.

### Results

#### Mind Wandering

Response variability to the eight mind wandering probes was again extremely limited. Only 11 total responses to the probes were either *the serial reaction task* or *something else.* All other participants responded to each response probe with *the video*, therefore, we did not include the mind wandering probes in any analyses.

#### Multiple Choice Questions

The mean proportion correct was .42 with a standard deviation of .25. Scores ranged from .00 to 1.00. Although low, a two-tailed, single-sample *t*-test indicated that performance on the quiz was above chance performance of .25, *t*(113) = 7.68, *p* < .001, *d* = .72.

#### Serial Reaction Task (SRT)

**Figure 3:**
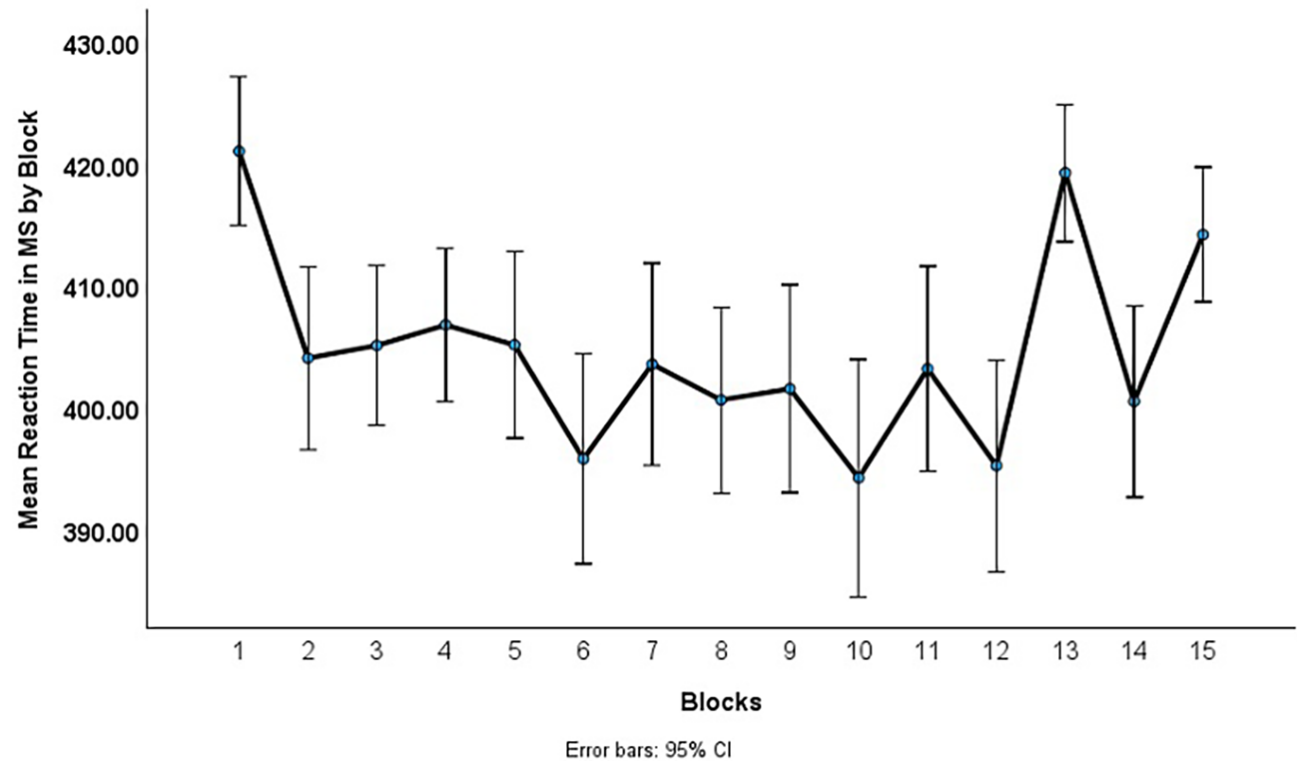
Mean Reaction Time by Block of Trials in Experiment 2

Figure 3 displays the mean RT for correct responses to Blocks 1–15. The mean RT improvement between Blocks 1 and 2 likely reflects practice effects. Because the linear function was the best fit to the data in Experiment 1, to measure rate of performance improvement, we calculated mean RTs per block and then fit a linear function across Blocks 2–12. The linear function fits the data *F*(1,1257) = 14.16, *R^2^* = .01, *p* < .001. Although small, the *R^2^* value of .01 indicates that 1% of the variance in reaction time is accounted for by time (blocks). Parameter estimates indicate an intercept of 413.55 and *b* = -1.23 (*t* = -3.76, *p* < .001) indicating that participants’ reaction time decreased significantly over the 12 blocks of trials. To verify that performance improvement was not only due to practice effects, we correlated RT differences between Blocks 2–3 with RT differences between Blocks 11–12. The correlation was not significant (*r* = .18, *p* = .08), suggesting that although we could not completely rule out practice effects, the linear fit parameters are valid for capturing overall performance improvement across blocks.

As in Experiment 1, the linear function parameter (*b*) was calculated for each individual participant. This linear function was correlated with proportion correct on the multiple-choice items. The correlation was not significant (*r* = .11, *p* = .29) suggesting that whether participants were paying attention to the video lecture they were implicitly learning the sequence presented in the SRT.

To examine whether implicit learning of the stimulus sequence, rather than simple practice effects for key presses, was responsible for RT improvement, a repeated measures ANOVA was conducted using Blocks 12–15. If participants RT was significantly longer for the non-sequence, random trials a slow down on Blocks 13 and 15 would be expected. The multivariate test was significant, Wilks λ = .77, *F*(3,98) = 9.53, *p* < .001. Within subjects contrasts between blocks indicate that participants were significantly slower on Block 13 than Block 12, *F*(1,100) = 24.69, *p* < .001, and significantly slower on Block 15 than Block 14, *F*(1,100) = 6.70, *p* = .011, suggesting that participants did not have explicit knowledge of the sequence.

#### Sequence Generation Task

In the sequence generating task, we considered a participant to have explicit knowledge if they could correctly produce five or more successive positions of the sequence (Willingham, 1998). None of our participants met this criterion in the current study. The mean number of correctly produced successive positions was *M* = 1.33 (*SD* = 2.31) and a mode of 0.

### Discussion

The results of Experiment 2 suggest that participants implicitly learned the 12-items sequence. This was the case no matter the level of learning of the video content. Put differently, even those participants who paid attention to the video and scored well on the multiple-choice questions demonstrated improved performance on the SRT.

Examining the results of the SRT, a significant decrease in reaction time over 12 blocks was revealed, indicating an improvement in performance. It can be concluded that this improvement in performance was not solely due to practice effects, as suggested by correlational analyses and slower reaction times on non-sequence trials. The non-significant correlation between SRT performance and multiple-choice question scores suggests that sequence learning occurred in my experiment no matter the degree of attention the participant gave to the video lecture. Additionally, participants showed a lack of explicit understanding of the sequence, as demonstrated by their inability to accurately reproduce consecutive positions. Overall, the data collected in Experiment 2 highlights the implicit learning processes during the SRT, whether the participant was paying attention to the video lecture.

## General Discussion

The objectives of the current study were to replicate Brosowsky et al.’s (2021) findings that implicit learning takes place during episodes of mind wandering and to expand upon their findings by examining whether implicit learning can also take place while individuals engage in a concurrent attention-demanding task (explicit learning). In both experiments, it was observed that participants implicitly learned a sequence of 12 items, regardless of their understanding of the concurrent video content. Even those who appeared to more actively engage with the video and performed well on the multiple-choice questions showed improved performance on the implicit learning task (SRT).

We anticipated significant instances of mind wandering among participants. Given the seemingly mundane nature of the SRT task and intentionally uneventful video pairing, we expected participants to report instances of mind wandering. However, upon analyzing the data, only 8 out of 94 participants reported mind wandering in the first experiment, and 11 out of 111 participants in the second experiment. While this might initially appear as accurate reporting, it’s crucial to compare the reported mind wandering instances with participants’ scores on the explicit learning task. In the first experiment, participants averaged 43% on the multiple-choice questions based on the video, and in the second experiment, the average score was 42%. Given these failing scores alongside the meager number of reported instances of mind wandering, it can be inferred that most participants were not truthful in their responses to the probes. If participants were genuinely focused on the video, they should have theoretically performed better on the multiple-choice questions. We suspect that participants may have been apprehensive about potential repercussions, leading them to report paying attention to the video due to a social presentation bias. Since participants were told to pay attention to the video during the instructions they may have been wary of reporting that they were mind wandering. It is also possible that because the instructions were to attend to the video, participants did do their best to attend to the video.

An alternative explanation regarding the lack of mind wandering reported, is that participants did not mind wander as much as we anticipated. Regarding the low scores on the quiz, although low, the scores were indeed above chance suggesting that participant did, at least in part, pay attention to the video. At the same time, participants’ response time to the SRT did in fact decrease indicating implicit learning of the sequence. Therefore, it may well be that participants were engaged in the two concurrent tasks and thus did not mind wander. In either case, we cannot draw any conclusion regarding the replication of Brosowsky’s work, or the influence of mind wandering on implicit learning.

This does not limit our ability to draw conclusions regarding the multitasking aspect of the current study. Again, participants on average performed better that chance in both experiments on the quiz. The correlation between quiz performance and change is reaction time during the SRT was not significant. This indicates that the amount of engagement with the video did not impact implicit learning of the SRT sequence. We conclude that one can implicitly learn a sequence of stimuli while engaged in an attention demanding explicit task.

It is important to note that our findings may not generalize to other implicit learning tasks. The SRT is simple sequence task of which increased performance may be partially due to procedural learning. Other implicit learning tasks, such as artificial grammar learning, may require other processes not captured by the SRT and thus may lead to different results. It may also be difficult to generalize our results to other explicit learning tasks. The video lecture was essentially a passive listening task. Tasks that require greater engagement and cognitive resources may not produce the same results. Future research should vary the implicit learning task and the explicit task to further test these hypotheses.

### Conclusion

Two separate experiments were carefully designed and conducted to gather more information on the relationship between implicit and explicit learning. The research conducted also covered the topics of mind wandering and multitasking’s impact on implicit learning. We aimed to measure whether implicit learning occurred when participants reported mind wandering while a secondary task with explicit attentional demands was administered concurrently with the implicit task. We hypothesized that implicit learning would occur, even while the participant was engaged in an explicit learning task, and reporting that they were mind wandering. Overall, our hypothesis was correct, and our data support our conclusion that individuals can implicitly learn while engaged in an explicit task. In our experiment, participants were able to complete the serial reaction time task more quickly and accurately over a time, essentially learning the pattern implicitly even while they were engaged in an explicit task. In the second experiment, when the pattern was abruptly changed, there was a measurable spike in reaction time during the SRT task indicating that any sequence learning was implicit, not explicit or dependent on episodic memory for the sequence. Our results suggest that multitasking is possible if one of the tasks is explicit and one is implicit.
